# Autophagy in rat annulus fibrosus cells: evidence and possible implications

**DOI:** 10.1186/ar3443

**Published:** 2011-08-16

**Authors:** Chao Shen, Jun Yan, Lei-Sheng Jiang, Li-Yang Dai

**Affiliations:** 1 Department of Orthopedic Surgery, Xinhua Hospital, 1665 Kongjiang Road, 200092, Shanghai, China

**Keywords:** Intervertebral disc, Autophagy, Apoptosis, Interleukin-1β, Serum deprivation

## Abstract

**Introduction:**

Programmed cell death of intervertebral disc (IVD) cells plays an important role in IVD degeneration, but the role of autophagy, a closely related cell death event, in IVD cells has not been documented. The current study was designed to investigate the effect of interleukin (IL)-1β on the occurrence of autophagy of rat annulus fibrosus (AF) cells and the interrelationship between autophagy and apoptosis.

**Methods:**

Rat AF cells were isolated and exposed, in tissue cultures with or without serum, to IL-1β in different concentrations for 24 hours. Ultrastructural analysis, flow cytometry and lysosomal activity assessment were performed after the *in vitro *treatment to determine the presence and levels of autophagy. The mRNA expression of autophagy-related proteins (Beclin-1, Bcl-2 and microtubule associated protein 1 light chain 3 (LC3)) were evaluated using real-time PCR. 3-methyladenine (3-MA), a PI3K inhibitor, was used to determine the interaction between autophagy and apoptosis via the suppression of autophagy.

**Results:**

Autophagy was detected in rat AF cells under serum starvation condition by transmission electron microscopy. PCR and flow cytometry results showed that IL-1β enhanced the autophagy-induction effect of serum deprivation in a dose-dependent manner. However, IL-1β alone failed to induce autophagy in AF cells cultured without serum starvation. When autophagy was suppressed by 3-MA, the apoptosis incidence was increased. Serum supplement also partly reversed the autophagy incidence without affecting the apoptosis incidence in the same cells.

**Conclusions:**

IL-1β up-regulates serum deprivation-induced autophagy of AF cells in a dose-dependent manner. Autophagy may represent a protective mechanism against apoptosis in AF cells and IVD degeneration.

## Introduction

Intervertebral disc (IVD) degeneration, associated with aging, is the common cause of neck or back pain in adults and thus often leads to reduction in quality of life [1]. IVD degeneration is characterized with loss of water content, decrease in proteoglycan synthesis, disappropriate collagen synthesis (switching from collagen type II to collagen type I), and abnormal production of the matrix metalloproteinases (MMPs) and ADAMTS (a disintegrin and metalloproteinase with thrombospondin motifs) [2,3]. Studies have suggested that IVD degeneration is a cell-mediated pathogenic process [4-6]: the disc cells, known as nucleus pulposus (NP) and annulus fibrosus (AF) cells, experience disturbed equilibrium of extracellular matrix turnover and fail to maintain biological and mechanical integrity of the disc [7]. Therefore, the physiopathology of disc cells has been the area of central interest in IVD study.

The programmed cell death is believed to play an essential role in tissue homeostasis as well as the pathogenesis of IVD degeneration [8-10]. The evidence from clinical and animal model studies has suggested that loss of disc cellularity is associated with apoptosis during the process of IVD degeneration [11-13]. Therefore, treatment targeting programmed cell death interception will be a potential direction for retarding or preventing IVD degeneration. However, although significant progress has been made in understanding apoptosis that is involved in IVD degeneration, the underlying mechanisms are not well understood.

Autophagy (the terms autophagy and autophagic used hereinafter refer to macroautophagy), first described in the 1960s by Christian et al. [14], has been known to be another pathway of cellular death in addition to apoptosis. Studies have revealed that the phenomenon "autophagy" is associated with some degenerative diseases, such as Parkinson's, Alzheimer's, Huntington's, and Crohn's disease [15-17]. In addition, autophagy and apoptosis are closely associated in the pathological process of human diseases and share some molecular events and regulators [18,19]. Although excessive autophagy triggers another pattern of cellular death (type II programmed cell death), autophagy is linked with survival advantage of cells facing different stimuli, especially in tumor cells, as an adaptive cell response allowing the cell to survive otherwise lethal challenges [20]. Different autophagy-related genes (Atg) are involved in this process. Beclin-1 (also known as Atg6) and microtubule-associated protein 1 light chain 3 (also known as Atg8, LC3) are required for autophagosome formation, one of the important steps for autophagy [21,22]. They are commonly used as autophagic markers. Also, Bcl-2, an anti-apoptotic protein, has been found to be a Beclin-1-interacting protein, and to exert anti-autophagic function [17].

Proinflammatory cytokines are also reported to anticipate IVD degeneration [3,23,24]. There have been a few studies focusing on the interplay between programmed cellular death and proinflammatory cytokines, which contribute to IVD degeneration [5,10,25,26]. IVD degeneration is associated with local increases in IL-1β [3,27]. IL-1β is able to induce apoptosis through mitochondrial dysfunction and endoplasm reticulum stress [28-31]. In the previous study, we found that IL-1β could amplify the effect of serum deprivation on rat AF cell apoptosis [10]. Recent evidence also demonstrated that IL-1β could induce apoptosis via JNK activation and that the activation of JNK could upregulate the disassociation of Beclin-1 and Bcl-2 complexes, which are involved in autophagy [32]. However, no reports have documented the relation between autophagy and IL-1β in chondrocytes or fibrochondrocytes.

The current study was designed to investigate the effect of IL-1β on the occurrence of autophagy of rat AF cells cultured with or without serum supplement, and to delineate the possible relation of autophagy to apoptosis. We show that IL-1β induces and upregulates autophagy in AF cells under serum deprivation. We also find that blocking autophagy leads to the increase of apoptosis incidence in AF cells.

## Materials and methods

### Isolation and culture of AF cells

Rat AF tissue (from L1-L2, L2-L3, L3-L4, L4-L5) was obtained from 16 male Sprague-Dawley rats, aged six weeks. Experimental protocol was approved by our Animal Care and Use Committee. After the discs were excised, the NP and inner AF were carefully removed by a scalpel microscopically under aseptic condition. The outer AF tissue was washed and cut into 1 mm^3 ^fragments. The fragments of AF tissues were digested in a serum withdrawal media containing 0.4% pronase for 90 minutes at 37°C, and then transferred to DMEM/Ham's F-12 (DMEM/F-12, Gibco, Carlsbad, CA, USA) with 5% fetal bovine serum (FBS), containing 0.025% collagenase Type II and 0.01% hyaluronidase Type V (from sheep testes, Sigma, St. Louis, MO, USA) for another 12-hour digestion at 37°C in a gyratory shaker. Tissue debris was removed by passing through a 70 μm filter. The resulting cells were seeded in 60 mm tissue culture dishes and incubated in a combined solution of DMEM/F-12 media and 15% FBS at a 37°C, 5% CO_2 _environment. Finally, the primary-passage cells were harvested and replanted into appropriate culture plates. First-passage cells maintained in a monolayer were used throughout the experiments.

### Reagents and antibodies

The Lyso-Tracker kit, Alexafluor 594-labeled and Alexafluor 488-labeled secondary antibodies were purchased from Invitrogen (Carlsbad, CA, USA). LC-3 and Beclin-1 antibodies were obtained from Abcam (Cambridge, UK). Hoechst 33258, 3-methyladenine (3-MA), monodansylcadaverine (MDC) and collagenases were from Sigma-Aldrich (St. Louis, MO, USA). The cell culture reagents were purchased from Gibco. IL-1β was purchased from Peprotech (Rocky Hill, NJ, USA).

### Transmission electron microscopy

At room temperature cells were fixed in 0.1% glutaraldehyde in PBS (pH = 7.4) for two hours, postfixed in 1% osmium tetroxide in water for one hour, and then stained in 2% uranyl acetate in water for one hour in the dark. After dehydrated in an ascending series of ethanol, the samples were embedded in Durcopan ACM for six hours, cut into 80 nm sections. These sections were stained with uranyl acetate and lead citrate, and examined with a Zeiss EM900 transmission electron microscope (Gottingen, Germany).

### Immunofluorescence

To detect LC3 and Beclin-1 proteins in rat AF cells, cells were prepared at a density of 50,000 cells per well in a 24-well plate. Cells cultured in the 24-well plates were washed three times in PBS and fixed with 4% paraformaldehyde in PBS (pH 8.0) for 10 minutes, and then washed three times with PBS. The cells were then permeabilized with 0.25% Triton-X 100 in PBS for 15 minutes and washed three times in PBS. Antigenic sites were blocked in 5% bovine serum albumin in PBS for 25 minutes. The cells were incubated with either LC3 or Beclin-1 antibody at a dilution of 1:100 overnight at 4°C. Subsequently, the treated cells were washed in PBS three times and incubated with a fluorescein-labeled secondary antibody for one hour at room temperature. The cells were then washed in PBS three times for five minutes. Protein localization was visualized by a confocal microscopy (Olympus Fluoview, Tokyo, Japan).

### Detection of autolysosomal activity

Lysosomal activity was assessed using the LysoTracker kit (Invitrogen, Eugene, Oregon, USA). Cells plated at a density of 50,000 cells per well were starved of serum for different IL-1β concentrations. These cells were then incubated with LysoTracker Red (75 nM) for one hour at 37°C under appropriate growth conditions. Lysosomal activity was assessed using confocal microscopy.

### Detection of autophagy incidence by flow cytometry

Cells were sub-cultured in six-well plates at 2 × 10^5 ^cells per well with complete culture medium. After reaching 90% confluence, the medium was changed to DMEM/F-12 containing 1% FBS and antibiotics for 12 hours in order to synchronize the cells. After treatment with different conditions, the cells were incubated with 0.05 mM MDC in PBS at 37°C for 10 minutes and then washed four times with PBS. Intracellular MDC was measured by flow cytometry within 30 minutes after incubation.

### Autophagy-induction by IL-1β in AF cells

To determine whether IL-1β induces autophagy in AF cells, we treated cells with different concentrations of IL-1β with the serum supplement or serum withdrawal media. First-passage rat annular cells were cultured with 0% or 10% FBS supplement and stimulated with 0, 10, 20 or 50 ng/ml IL-1β for 12, 24 or 36 hours. Then cells were sent for assessment of the autophagy incidence by flow cytometry and lysosomal activity by confocal microscopy, respectively.

### Detection of apoptosis incidence by flow cytometry

Apoptosis incidence was detected by using the Annexin V-FITC apoptosis detection kit I (BD Pharmingen, San Diego, CA, USA). Briefly, cells that still attached to the plate as well as those present in the supernatant were collected together and re-suspended in one times binding buffer at a concentration of 1 × 10^6 ^cells per ml. A 100 μl sample of solution containing 1 × 10^5 ^cells was incubated with 5 μl of AnnexinV-FITC and 5 μl of propidium iodide for 15 minutes at room temperature in the dark, followed by addition of 400 μl of one times binding buffer. Samples were analyzed by a fluorescence-activated cell sorter (Beckman Coulter, Miami, FL, USA) within one hour. Apoptotic cells, including those staining positive for Annexin V-FITC and negative for propidium iodide and those that were double positive, were counted and represented as a percentage of the total cell count.

### Detection of apoptotic cells by Hoechst 33258 staining

Apoptotic cells were detected by using the Hoechst 33258 staining (Beyotime, Haimen, China). The AF cells were prepared at a density of 50,000 cells per well in a 24-well plate. After treatment with 3-MA, the cells were fixed with 4% paraformaldehyde for 15 minutes, washed with PBS for three times and stained with 2 μg/ml Hoechst 33258 (Sigma, St. Louis, MO, USA) in Hank's balanced salt solution for five minutes. Morphologic changes in apoptotic nuclei were evaluated under a fluorescence microscope (Olympus Fluoview, Tokyo, Japan) with excitation at 350 nm and emission at 460 nm.

### Rescue effects of 10% FBS on autophagy incidence

The first-passage AF cells were placed in six-well plates at 2 × 10^5 ^cells per well. After serum starvation for 24 hours, the autophagy incidence was measured by fluorescence photometry with MDC positive staining in half of the AF cells. The rest of cells were treated with 10% FBS for six hours and examined for the autophagy incidence again by flow cytometry.

### Effect of 3-MA upon interplay between autophagy and apoptosis in AF cells

First-passage rat AF cells were incubated in serum withdrawal media with 20 ng/ml IL-1β for 24 hours in the presence or absence of 3-MA, a specific autophagy inhibitor of through PI3K/Akt/mTOR pathway, was used to investigate the interaction between autophagy and apoptosis. The autophagy and apoptosis incidence of AF cells were recorded.

### Real-time PCR

After first-passage AF cells were stimulated with different concentration of IL-1β with or without serum supplement, the RNA of cells was isolated using Trizol reagent (Invitrogen, Carlsbad, CA, USA). The expression of Beclin-1, LC3 and Bcl-2 genes was determined by real-time PCR using SYBR Premix Ex Taq (Takara, Shiga, Japan) and an ABI Prism 7500 sequence detection system (Applied Biosystems, Foster City, CA, USA) with the following primers: 5'-TGAACCGGCATCTGCACAC-3' and 5'-CGTCTTCAGAGACAGCCAGGAG-3' for Bcl-2 (116 bp); 5'-CATGCCGTCCGAGAAGACCT-3' and 5'-GATGAGCCGGACATCTTCCACT-3' for LC3/Atg8 (70 bp); 5'-TTCAAGATCCTGGACCGAGTGAC-3' and 5'AGACACCATCCTGGCGAGTTTC-3' for Beclin-1/Atg6 (142 bp). The reaction mixture was amplified at 50°C for two minutes and 95°C for 30 seconds and then 40 cycles of 95°C for five seconds followed by 60°C for 34 seconds. The optimal concentrations of primers and templates used in each reaction were established according to the standard curve created before the reaction and corresponding to the nearly 100% efficiency of the reaction. The fold-change in gene expression relative to the control was calculated by 2-ΔΔCT.

### Statistical analysis

Results were expressed as mean ± standard deviation. Statistical analyses were performed using the SPSS 11.5 statistical software program (SPSS Inc., Chicago, IL, USA). The means of mRNA relative folds, autophagy incidences among groups receiving identical concentrations of IL-1β and identical concentrations of FBS for the same experimental duration were compared by two-way repeated measure analysis of variance (ANOVA) with a *post-hoc *Student-Newman-Keuls test. Data regarding 3-MA effects on autophagy and apoptosis for cells treated with the same concentration of IL-1β with and without serum supplement as well as the results for 10% FBS effects on autophagy were analyzed using paired *t *test. A *P *value less than 0.01 was considered statistically significant.

## Results

### Serum-starvation-induced autophagy in isolated rat AF cells

**Figure 1:**
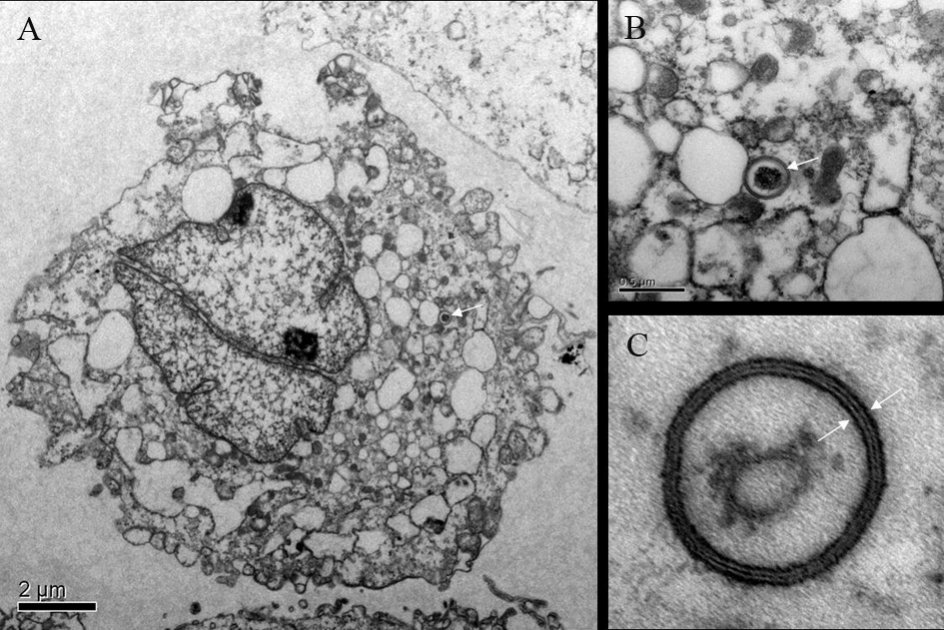
**Evaluation of autophagy in the AF cells under electronic microscope**. Electron micrographs of annulus fibrosus (AF) cell after 24 hours of starvation. **(a) **AF cell in autophagy, observed by electronic microscopy in 8,000×. **(b and c) **A typical autophagosome in 50,000× and 150,000×. Double-limiting membrane could be observed in the autophagosome (arrowheads). The contents of the autophagosome might be a cellular organelle, mitochondria or RNA.

**Figure 2:**
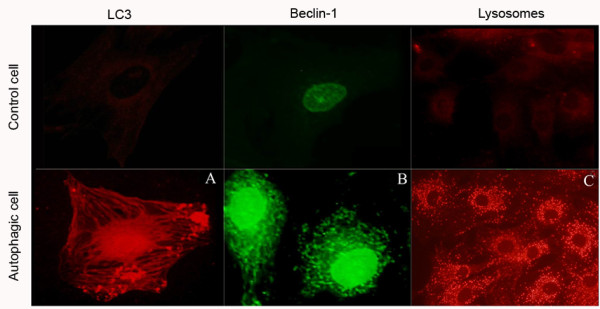
**Detection of autophagy-related proteins and lysosomes**. Autophagy-related proteins, Beclin-1 and microtubule associated protein 1 light chain 3 (LC3) were assessed by the immunofluorescence method. **(a) **LC3 was stained by Alexafluor 594-labeled second antibody. **(b) **Beclin-1 was stained with Alexafluor 488-labeled second antibody. **(c) **The activity of lysosomes was assessed using a Lyso-Tracker kit. The activated lysosomes were shown in the cytoplasm. The annulus fibrosus (AF) cells cultured in 10% fetal bovine serum (FBS) were used as controls.

To verify whether autophagy occurs in rat AF cells, we used electronic microscopy to visualize autophagy vesicles in the cytoplasm. As expected, autophagosomes were detected in AF cells after a stimulation of starvation for 24 hours (Figure 1). Microtubule-associated protein LC3 (also known as Atg8), a well-validated biomarker of autophagy, was detected readily in the cytoplasm of the AF cells following 24 hours of serum starvation (Figure 2a). In addition, Beclin-1/Atg6, another key autophagy protein, was also detected in the AF cells (Figure 2b). Autolysosomes were rich in the cytoplasm as shown by the Lyso-Tracker after a 24-hour starvation (Figure 2c).

### Effect of IL-1β on serum-deprivation-induced autophagy in AF cells

We conducted two sets of experiments to evaluate whether IL-1β could induce autophagy in rat AF cells. In the first set of experiments, all AF cells were cultured with 10% FBS. Our preliminary experiments showed that autophagy of AF cells could hardly be detected during the first 12 hours culture in medium with 10% FBS. A parallel increase in the rate of autophagy and apoptosis was observed after 48-hour culture with 10% FBS. Thus, a period of 24-hours was selected in order to avoid masking the effect caused by IL-1β stimulation. In this period, autophagy incidence was relatively low and increased gradually over time. The increase in concentrations of IL-1β led to a slight increase in autophagy incidence which was not statistically different compared with vehicle control. These results suggest that IL-1β could not induce autophagy in AF cells cultured with 10% FBS.

**Figure 3:**
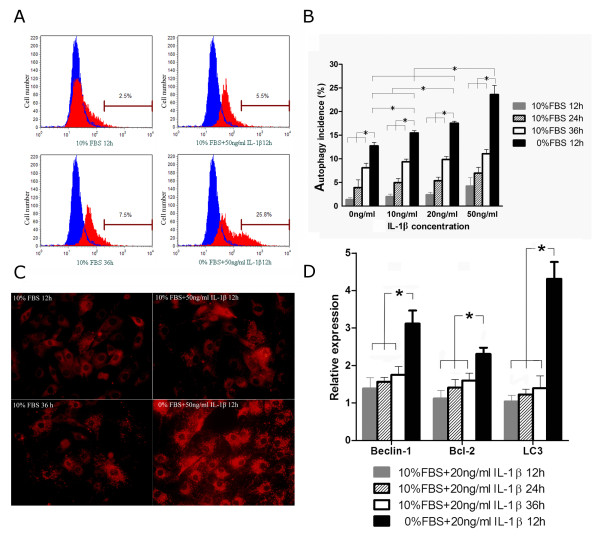
**Autophagy incidence under different IL-1β concentrations and serum supplement**. **(a) **Representative graphs obtained by flow cytometry analysis after monodansylcadaverine (MDC) staining. The data showed that the levels of autophagy were relative low when the cells were cultured with 10% fetal bovine serum (FBS). However, with serum withdrawal media, the incidence of autophagy obviously increased. **(b) **The autophagy incidences of rat annulus fibrosus (AF) cells cultured with or without serum supplement and stimulated with different concentrations of IL-1β. All data are presented as the mean ± standard deviation (SD). *P < 0.01. **(c) **Activity of cytoplasm lysosome, assessed using a Lyso-Tracker kit. The results indicated that IL-1β did not change the activity of lysosome when rat AF cells were incubated with 10% FBS. Conversely, serum deprivation obviously increased the activity of lysosome. **(d) **mRNA expression of autophagy-related genes in rat AF cells by real-time PCR analysis when treated with or without serum supplement and IL-1β (20 ng/ml). The mRNA levels of AF cells which were cultured in 10% FBS without IL-1β were used as controls. All data are presented as the mean ± SD. *P < 0.01. Serum deprivation obviously increased the mRNA expression levels of Beclin-1, Bcl-2 and microtubule associated protein 1 light chain 3 (LC3) after 12 hours.

In the second set of experiments, all AF cells were cultured in the serum-free media. There was no significant increase in the autophagy incidence AF cells cultured for six hours under serum starvation conditions, whereas there was a significant increase in both autophagy and apoptosis incidence of cells after 24-hour serum starvation. Thus, we examined autophagy incidence in rat AF cells after 12 hours of serum starvation. The results showed that the 12-hour serum deprivation resulted autophagy in around 13% of the rat AF cells. Adding 10 ng/ml or more of IL-1β significantly augmented the autophagy incidence AF cells as quantified with flow cytometry (Figures 3a and 3b). At the concentration of 10 ng/ml, IL-1β induced an increase of 1.2 fold of the autophagy incidence (autophagy incidence = 15.5 ± 0.47%). The concentration of 20 ng/ml and 50 ng/ml induced an increase of 1.38 and 1.85 fold of autophagy incidence in AF cellls (autophagy incidence = 17.5 ± 0.42% and 23.6 ± 1.64%). The results showed that autophagy incidence was gradually increased over time but IL-1β did not induce autophagy when the AF cells were cultured with 10% FBS. In contrast, serum deprivation easily induced autophagy in AF cells. Moreover, IL-1β upregulated the autophagic effect of serum deprivation in a dose-dependent manner.

In order to further corroborate the findings in our flow cytometry studies, we next examined lysosome activity and mRNA expression of autophagy-related genes (Beclin-1, Bcl-2, and LC3). As shown in Figure 3c, the density of Lyso-Tracker staining did not change when the AF cells were cultured with 10% FBS. However, the density of Lyso-Tracker was significantly increased when cells were serum deprived for 12 hours, compared with that of the cells cultured with 10% FBS. Based upon the results of the preliminary study, IL-β at the concentration of 20 ng/ml was chosen to examine mRNA expression of Beclin-1, Bcl-2, and LC3. Consistent with the quantification of the rate of autophagy, serum deprivation induced a significant increase in Beclin-1, Bcl-2, and LC3 expression in AF cells, which was not observed over time with serum supplementation (Figure 3d). These results suggests that IL-1β is not capable of inducing autophagy in AF cells by itself, but it can significantly potentiate autophagy under serum starvation at least.

### The autophagy in AF cells is partially rescued by 10% FBS treatment

**Figure 4:**
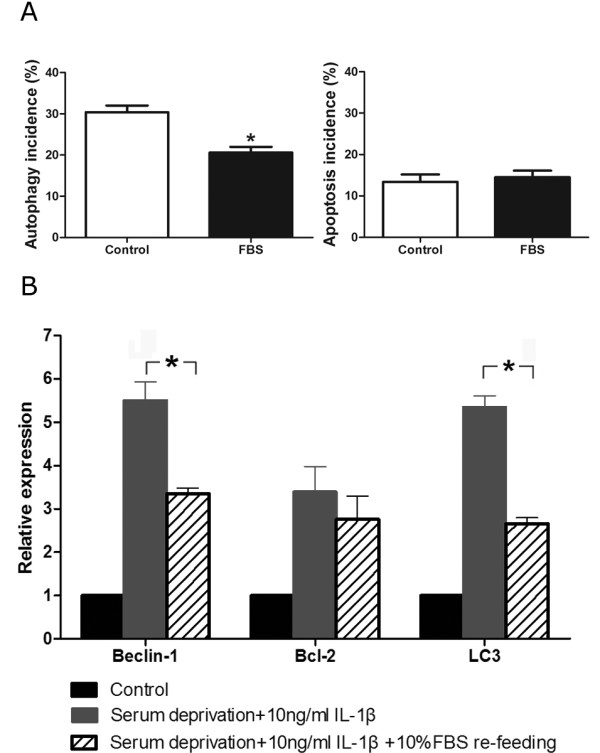
**Autophagy incidence is partially rescued by 10% FBS**. **(a) **The incidence of autophagy annulus fibrosus (AF) cells after re-fed with 10% fetal bovine serum (FBS) for six hours. The cells were cultured in serum deprivation media with 10 ng/ml IL-1β for 24 hours to induce autophagy. Then the cells were re-fed with 10% FBS for six hours and the autophagy incidence was significantly decreased, whereas the apoptosis incidence showed no significant change, indicating that autophagy in the AF cells was rescued to some degree. All data are presented as the mean ± standard deviation (SD). *P < 0.01. **(b) **mRNA expression of autophagy-related genes in rat AF cells by real-time PCR analysis with or without serum supplementation. All data are presented as the mean ± SD. The mRNA levels of AF cells which were cultured in 10% FBS without IL-1β were used as controls. After re-fed with 10% FBS for six hours, the mRNA expression levels of Beclin-1 and microtubule associated protein 1 light chain 3 (LC3) decreased significantly. *P < 0.01.

To determine whether autophagy could be rescued, we evaluated the impact of nutrient supplementation on the fate of autophagy in AF cells. The AF cells were first cultured in serum withdrawal media with IL-1β at the concentration of 10 ng/ml for 24 hours to induce autophagy. According to the results of our preliminary experiment, re-feeding the cells with 10% FBS for three hours significantly reduced the autophagy incidence and in turn led to an increase in the total number of viable cells 12 hours later. Therefore, we measured autophagy incidence six hours after 10% FBS application after they had been exposed to serum starvation plus IL-1β. The autophagy incidence was reduced from 30.37 ± 0.95% to 20.60 ± 0.79% after cells were re-fed with 10% FBS for six hours. There was no increase in apoptosis incidence in those AF cells (Figure 4a). The mRNA expression of Beclin-1, Bcl-2, and LC3 was also evaluated. As expected, the levels of Beclin-1 and LC3 mRNA were significantly reduced by 10% FBS (Figure 4b). The results were consistent with those shown by flow cytometry (Figure 4a). All these findings indicate that autophagy can be rescued to some degree when cells are cultured with nutrient supplementation.

### Apoptosis increases with autophagy inhibition

**Figure 5:**
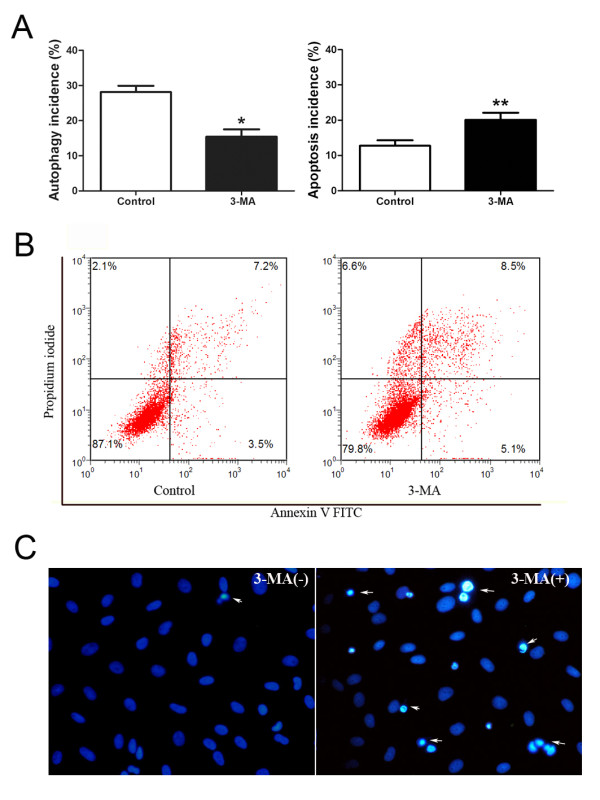
**Interrelationship between autophagy and apoptosis in AF cells**. Autophagy incidence was obtained by monodansylcadaverine (MDC) staining and apoptosis incidence was assessed by double staining with Annexin V-FITC and propidium iodide. **(a) **The autophagy incidence and the apoptosis incidence were observed after the cells were treated with the autophagy inhibitor, 3-methyladenine (3-MA). The data are presented as the mean ± standard deviation (SD). *P < 0.01, ** *P *< 0.001. **(b) **Representative graphs obtained by flow cytometry analysis after double staining with Annexin V-FITC and propidium iodide. Undamaged cells were stained with negative Annexin V-FITC/PI (bottom left quadrant). After application of 3-MA, the incidence of apoptotic cells increased. **(c) **Morphologic changes in apoptotic rat annulus fibrosus (AF) cells by Hochest staining. Phase-contrast photomicrograph of rat annular cells cultured in serum-free medium stimulated with IL-1β and 3-MA for 24 hours. Apoptotic cells were characterized by brightly stained with Hoechst 33258 (arrowheads). The results indicate the interrelationship between autophagy and apoptosis. When autophagy is inhibited, the apoptosis incidence is significantly increased.

To investigate the interplay between autophagy and apoptosis in the AF cells, cells were treated with 3-MA (5 mM), an autophagy inhibitor. Treatment with 3-MA significantly diminished the autophagy incidence, which was induced by 24-hour IL-1β treatment under serum deprivation in AF cells (Figure 5a). In contrast, 3-MA significantly increased the apoptosis incidence in AF cells under this experimental condition, shown by flow cytometry (Figure 5a and 5b). The Hochest staining of apoptotic cells was also observed by using a phase-contrast microscopy (Figure 5C5c). The results suggest that the inhibition of autophagy does trigger apoptosis in the AF cells.

## Discussion

In the current study, we confirmed that, for the first time, autophagy takes place in AF cells as shown by evidence from electronic microscopy and immunofluorescence examination. To the best of our knowledge, this is the first report of autophagy in AF cells. Our results suggest that IL-1β does not induce autophagy in AF cells by itself, but it augments the autophagy induced by serum deprivation. No morphological changes were observed by microscopy during the autophagy process. Our study also shows that the inhibition of autophagy in AF cells is accompanied by a significant increase in the apoptosis incidence. On the other hand, autophagic AF cells could be rescued by re-feeding with FBS. These results demonstrate that autophagy partially protects AF cells from apoptosis, when AF cells face the stimulation of IL-1β and serum deprivation. During IVD degeneration, both the annulus fibrosus and the nucleus suffer from insufficient nutrient supply and local increase of IL-1β [3,27]. Thus, these findings indicate that autophagy may play an important role in the pathogenesis of IVD degeneration.

Recent studies have documented autophagy in articular cartilage. Bohensky et al. [33], based on their experiments, suggested that autophagy could be induced in chondrocytes and regulated by hypoxia-inducible factor family. Almonte-Becerril et al. [34] concluded that both apoptosis and autophagy were observed in chondrocytes during pathological process of osteoarthritis (OA). Caramés et al. [35] used a mouse OA model and found that Atg gene and proteins, which are crucial for autophagosome formation, are strongly expressed in OA chondrocytes and decreased together with the reduction of glycosaminoglycans. Thus, they suggested that reduction of autophagy might play an important role in the development of OA. Based upon these results and our findings, we suggest that autophagy should be involved in IVD degeneration as the clearing system because age-related IVD degeneration is a process characterized by a progressive accumulation of damaged macromolecules that reduces the capacity of the IVD to self-renew when the disc undergoes decreased anabolism and/or increased catabolism [36].

Pro-inflammatory cytokines are known to be involved in IVD degeneration [3,23,24] by interacting with MMPs and degrading the extracellular matrix of IVD cells, which are important in maintaining the normal spine function. IL-1β can induce mitochondrial dysfunction and apoptosis in chondrocytes [28-30]. Lopez-Armada et al. [30] reported that a 48-hour treatment with IL-1β-induced apoptosis in human chondrocytes incubated without FBS. However, Oliver et al. [37] reported that stimulation by 1 ng/ml IL-1β administration does not induce apoptosis of human costal chondrocytes cultured with 10% FBS for 24 hours. Our previous study [10] also showed that IL-1β did not induce rat AF cell apoptosis when cultured in medium containing 10% FBS. However, when cells were cultured with serum deprivation for 24 hours, apoptosis was detected. These findings suggest that Il-1β alone is not the stimulus sufficient to induce disc cell apoptosis.

Similarly, we failed to induce autophagy of AF cells by administrating IL-1β with no serum deprivation in this study. The role of IL-1β as shown by the data from the study seems to be just augmenting the autophagy induction effect of serum deprivation. This argument is also supported by our findings that the autophagy incidence in AF cells is reversed partially by supplying FBS. All these results indicate that serum deprivation is the common factor of apoptosis or autophagy in the IVD. Programmed cell death occurs characterized with nutrient consumption and growth factor loss in a time-dependent manner [38-41]. Therefore, the IVD experiences a decrease in nutrient supplement and increase in inflammatory cytokines production during aging [3,42,43]. Improvement of nutrient supply to the degenerative disc would be one therapeutic modality.

We also demonstrated interplay between autophagy and apoptosis in AF cells. AF cells treated with 3-MA, the autophagy inhibitor, showed a significant decrease in the autophagy incidence after a 24-hour stimulation of IL-1β under serum deprivation, whereas an increase in the apoptosis incidence was noted, thus indicating that the inhibition of autophagy has triggered apoptosis in the AF cells. Autophagy can be a cell survival response to different apoptosis inducers such as nutrient deprivation. Induction of autophagy has been proved to be protective from apoptosis in the aging process and age-related degenerative diseases [44]. The results of this study may explain our previous finding that the increase in the incidence of apoptosis in AF cells occurs until 24 hours after serum deprivation [6]. We believe that autophagic response may contribute to the delay in apoptosis and IVD degeneration.

The results of this study need to be further verified. Heraud et al. [45] found that chondrocyte apoptosis could be induced by IL-1β when cells were cultured without serum deprivation. Nevertheless, all the chondrocytes in their experiment were isolated from osteoarthritic or healthy elderly patients with ages ranging from 67 to 87 years. There may be intrinsic differences that distinguish these cells from normal articulate chondrocytes. In the current study only cells from rat outer AF were used so that the homogeneity of cells cultured *in vitro *would be maintained and interactions between cell types excluded. Rannou et al. [46] showed that AF is stimulated by IL-1β *in vitro *to produce factors implicated in degenerative processes but is less responsive to IL-1β-induced apoptosis than articular chondrocytes. Therefore, further studies should be undertaken to compare the role of autophagy between AF cells and NP cells and articular cartilage cells, although they all share similar biological features as chondrocyte-like cells. The study may be limited by the types of cells we used: we used only AF cells in the current study, but both NP and AF cells are involved in the degeneration process of IVD. We just obtained cells from outer AF tissue for 2D culture, and no phenotypic changes of these cells were assessed. In fact AF cells under monolayer culture might experience changes in their phenotypes characterized by increased expression of collagen type I and decreased expression of collagen type II [47]. The study may be also limited in donor selection of IVD cells: we harvested nondegenerative cells from healthy rats and the role of autophagy should be examined in different experimental and clinical settings.

## Conclusions

In summary, we have shown, for the first time, that autophagy can be induced and up-regulated by IL-1β with serum deprivation in rat AF cells. When autophagy is inhibited by 3-MA, apoptosis increases in AF cells. However, FBS can also inhibit autophagy and promote survival of AF cells. Our results indicate that autophagy may be involved in IVD degeneration and that inhibition of autophagy by nutrient supplement may be of therapeutic implication to intervening for IVD degeneration.

## Abbreviations

3-MA: 3-methyladenine; AF: annulus fibrosus; ANOVA: analysis of variance; Atg: autophagy-related genes; DMEM: Dulbecco's modified Eagle medium; FBS: fetal bovine serum; IL-1β: interleukin-1β; IVD: intervertebral disc degeneration; LC3: microtubule-associated protein 1 light chain 3; MDC: monodansylcadaverine; MMPs: matrix metalloproteinases; NP: nucleus pulposus; OA: osteoarthritis; PBS: phosphate buffered saline.

## Competing interests

The authors declare that they have no competing interests.

## Authors' contributions

CS participated in the study conception and design and participated in manuscript preparation and revision. LYD participated in the study conception and design and participated in manuscript preparation and revision. JY participated in carried out the experimental work the data collection and analysis and participated in manuscript preparation and revision. LSJ participated in manuscript preparation and revision. All authors read and approved the final manuscript.

## Acknowledgements

This study was supported by the National Natural Science Foundation of China (U1032001, 81000793, 81071500).
